# Dental skill mix: a cross-sectional analysis of delegation practices between dental and dental hygiene-therapy students involved in team training in the South of England

**DOI:** 10.1186/1478-4491-12-65

**Published:** 2014-11-18

**Authors:** Kristina L Wanyonyi, David R Radford, Jennifer E Gallagher

**Affiliations:** King’s College London Dental Institute, Division of Population and Patient Health, Bessemer Road, London, SE5 9RS UK; Teaching Division, King’s College London Dental Institute, Guys Tower, Guys Hospital, St Thomas Street, London, SE1 9RT UK; University of Portsmouth Dental Academy, William Beatty Building, Hampshire Terrace, Portsmouth, Hampshire, PO1 2QG UK

## Abstract

**Background:**

Research suggests that health professionals who have trained together have a better understanding of one another’s scope of practice and are thus equipped for teamwork during their professional careers. Dental hygiene-therapists (DHTs) are mid-level providers that can deliver routine care working alongside dentists. This study examines patterns of delegation (selected tasks and patients) by dental students to DHT students training together in an integrated team.

**Methods:**

A retrospective sample of patient data (n = 2,063) was extracted from a patient management system showing the treatment activities of two student cohorts (dental and DHT) involved in team training in a primary care setting in the South of England over two academic years. The data extracted included key procedures delegated by dental students to DHT students coded by skill-mix of operator (e.g., fissure sealants, restorations, paediatric extractions) and patient demography. *χ*^2^ tests were conducted to investigate the relationship between delegation and patient age group, gender, smoking status, payment-exemption status, and social deprivation.

**Results:**

A total of 2,063 patients managed during this period received treatments that could be undertaken by either student type; in total, they received 14,996 treatment procedures. The treatments most commonly delegated were fissure sealants (90%) and restorations (51%); whilst the least delegated were paediatric extractions (2%). Over half of these patients (55%) had at least one instance of delegation from a dental to a DHT student. Associations were found between delegation and patient age group and smoking status (*P* <0.001). Children under 18 years old had a higher level of delegation (86%) compared with adults of working age (50%) and patients aged 65 years and over (56%). A higher proportion of smokers had been delegated compared with non-smokers (45% cf. 26%; *P* <0.001).

**Conclusions:**

The findings suggest that delegation of care to DHT students training as a team with dental students, involved significantly greater experience in treating children and adult smokers, and providing preventive rather than invasive care in this integrated educational and primary care setting. The implications for their contribution to dentistry and the dental team are discussed, along with recommendations for primary care data recording.

## Background

The current position paper of the World Health Organization on scaling up the health workforce considers inter-professional training as an essential step in the development of a collaborative health workforce [1]. In both developed and developing countries, the concept of collaborative practice within the dental team is encouraged, particularly through task sharing and wider use of mid-level dental providers [2–5]. There are several cadres of mid-level dental providers; collectively, they are often referred to as dental care professionals (DCPs) or dental auxiliaries, and include dental hygienists, dental nurses, orthodontic therapists, dental hygiene-therapists, clinical dental technicians, and dental technicians [6].

In recent times, the developing role of dental hygiene and dental therapists has gained wide interest [7–9]. This is because many see potential for improved capacity of dental services through task sharing between dentists and other members of the dental team due to their overlap in skills [10, 11]. In the UK, there has been a recent move to train dually qualified dental hygiene-therapists [12]. The scope of practice of a dental hygienist is within that of a dental therapist [13], and, when qualified, dental therapists may register and work as a dental hygienist and as a dental therapist. The equivalent personnel who have training to the level of a dental therapist are referred to differently between countries; for example, dental therapists (New Zealand, Malaysia, and USA) [10]; oral health technicians (Brazil) [14]; oral health therapists (Holland) [10]; and dental hygiene-therapists or dental therapists (UK) [6]. The development of their role or scope of practice and the regulation of their practice also vary between countries. These variations mainly revolve around the ‘level of autonomy’ and ‘scope of practice’ [14, 15]. These personnel commonly provide routine care that includes scaling, filling cavities, preventive care, and extraction of children’s teeth [16].

In The Netherlands and the UK, the training of hygienists, who mainly work in clinical prevention, has been expanded into dually qualified hygiene-therapists [10, 12]. In Scandinavian countries, where there are hygienists but no hygiene-therapists, researchers have suggested that the training of hygienists should be expanded to provide them with sufficient skills and the confidence to carry out a greater variety of clinical treatment measures in the future [2]. The rationale behind these changes appears to be to give more time for dentists to cope with increased demand for complex treatments and care for medically compromised patients; these factors are associated with the changing demands and increasingly ageing populations whilst also recognising general improvements in oral health and that some more simple tasks may be delegated to other members of the dental team [10, 17, 18].

In the UK, the General Dental Council (GDC), as the regulating body, has outlined the roles and responsibilities of all dental professionals, including dental therapists in the GDC ‘Scope of Practice’ guidance [13], and they have recently approved the concept of ‘Direct access’ for patients to dental hygienists and dental therapists [19]. This regulation falls in line with other contemporary government policies that have encouraged a team approach to primary dental care services in order to meet the changing demands on dental services [20]. This follows several decades of documents calling for wider use of DCPs, task sharing, and skill mix [21]. Despite such lengthy support, DCPs, particularly those holding a dental therapy qualification, appear to be underutilised [12, 15].

Research in the UK to ascertain the reasons for the underutilisation of dental hygiene-therapists has revealed a lack of understanding and misconceptions over their scope of practice amongst dentists [22–26]. Evidence from Brazil suggests that lack of autonomy and credibility with the public has led to challenges in developing the role of oral health technicians [14]. In the USA, whilst dental therapists have developed in many states, they have continued to face opposition from national and state dental associations [27]. In Scandinavia, developing the scope of practice of hygienists also remains an issue of debate [2].

The economic implications of using dental hygiene-therapists have also been the subject of debate [28, 29]. Sun et al. [29] found that although some practices have found ways to incorporate dental hygiene-therapists in their practices, practice principals find it challenging to evaluate their contributions and plan for payments, because there is lack of management information on their productivity.

In regards to acceptability of dental hygiene-therapists, there is evidence that the public and patients in the UK find them acceptable, but knowledge of their roles is unclear and further education of the public is suggested [30, 31]. As the number of dental hygienists-therapists in training has expanded, consideration is increasingly being given to developing multi-professional training. Ross et al. [10] suggest that dental students who have been trained together with DCPs have a better understanding of DCP’s scope of practice than those who have not. It is therefore important that there is a clear understanding of respective roles of the members of the dental team to further develop skill mix in practice.

As the agenda to promote teamwork, skill mix, and task delegation continues, a clear call for more empirical data on the contribution dental hygiene-therapists make to clinical care has been made [11, 29]. Apart from one observational study in primary dental care by Evans et al. [11] in general dental practices in South Wales, little is known about patient delegation within the state funded health system in the UK and there is no published information on what happens when dental and dental hygiene-therapy students train together. This study seeks to inform this knowledge gap by contributing findings that will be useful in aiding the understanding of how delegation can work within the dental team in training and analysing data from a patient management system that is common to primary dental care nationally.

This research aimed to examine the activities of a team of dental and dental hygiene-therapy students training together in an integrated team-training primary care environment in the South of England, where barriers to delegation placed by the payment system do not exist. In particular, the analysis focuses on the patterns of delegation of tasks from final year dental students to dental hygiene-therapy students. The dental students, who are under the supervision of tutors, examine and formulate care plans that may involve delegation to dental hygiene-therapy students. This is an initial approach to inform a quantitative knowledge gap, using statistical analysis to show magnitude and distribution of delegation and by itself does not answer all questions about delegation, which would include wider elements, features, facilitators, and hindering factors of delegation.

## Methods

The facility at the centre of this research is the University of Portsmouth Dental Academy (UPDA) in the South of England. It is a primary care dental training centre opened in September 2010 to integrate education and training of dental students on outreach training from King’s College London with DCPs (dental hygiene-therapy and dental nursing students) training in Portsmouth. The facility was expanded in order to improve both service capacity for the surrounding community and enhance teamwork training in dentistry, having previously only hosted the training of DCPs in any given week, 20 dental students worked three and a half clinical days with 2^nd^ and 3^rd^ year dental hygiene-therapy students (24 per cohort) undertaking two days of clinical work per student.

This research was conducted using retrospective cross-sectional patient data obtained from the electronic patient management system at UPDA. The findings presented here form part of a wider body of research that looks into case mix and skill mix at UPDA as well as access to dental care [32]. Ethical Approval was given by NRES Committee Fulham REC: Reference No. 11/LO/1138 Protocol No. NTMHWMOV3 and NHS Portsmouth R&D Committee Reference No. SSPS/05/11.

The data comprised patient demography and treatment activity in the first 2 years of team training (1 September 2010 to 31 August 2012). Clinical activity included treatment item codes, which indicated the performer of the treatment (dental student or dental hygiene-therapy student). For example, an amalgam restoration would be coded either amalgam restoration for dental student [Amalgam filling-DS] or amalgam restoration for dental hygiene-therapy student [Amalgam filling-HTS], depending on the type of student. This coding structure was part of the patient management software modified by UPDA. Dental students undertook patient assessments and treatment planning including whether or not a treatment should be delegated to a dental hygiene-therapy student, and coded the care accordingly. Dental students had the freedom to delegate tasks within the dental hygiene-therapists scope of practice.

All data on patients who had one or more procedures, labelled by provider of care, were eligible for analysis (n = 2,063). These included paediatric tooth extractions (related to disease or exfoliation), restorations, pulpotomies (endodontic treatment on primary teeth), fissure sealants, and urgent care. Other less complex clinical items which may be delegated were not coded by provider of care within the patient management system, most notably scale and polish and fluoride varnish, and thus were not available for skill mix analysis. *χ*^2^ tests were applied to examine the relationship between delegation to dental hygiene-therapy students and patient socio-demographic characteristics; this included patient ethnicity, age, gender, payment status, smoking status, and quintile of deprivation.

Age was analysed in age-groups. First, in three categories ‘under 18 years’, ‘18–64 years’ (working age adults), and ‘over 64 years’. A further analysis of the distribution of delegation by age was undertaken using the 11 National Health Service (NHS) age-groups (0–2 years, 3–5 years, 6–12 years, 13–17 years, 18–24 years, 25–34 years, 35–44 years, 45–54 years, 55–64 years, 65–74 years, Over 75 years).

Payment status identifies whether a patient is exempt from charges or not within the NHS system, albeit that in this educational setting charges did not apply. Adults of different social circumstances, for example, receiving unemployment benefit, are exempt from payment [33]. All children are automatically exempt from payment in line with the policy in state funded dental care in England; therefore, only adult payment exemption status was analysed in this study. Smoking status and whether a patient was signposted for smoking cessation are automatically collected in the patient management system as clinicians are required to collect this information as part of the payment contract and in support of delivery of preventative care. Quintiles of deprivation were calculated based on the Index of Multiple Deprivation (IMD) score, a measure that provides a relative measure of deprivation at small area level across England [34].

## Results

### Patient characteristics and delegation

There were 2,063 patients and a total of 14,996 treatment procedures in the study data set; 55% (1,134) of patients had evidence of at least one instance of delegation. There were statistically significant relationships between delegation and patient characteristics (Table 1). Younger patients were delegated to dental hygiene-therapy students at a higher rate than other groups (*P* <0.001), with the majority of patients under the age of 18 years (86%) having been delegated at least once, compared with 54% of older adults (≥64 years) and 50% of working age adults (18–64 years). A higher proportion of smokers had been delegated, compared with non-smokers (45% cf. 26%; *P* <0.001). No significant difference was found in the proportion of patients delegated by gender, quintile of deprivation, or payment status.

**Table 1:** **Differences in delegation rate by patient socio-demography**

	Row total	No delegation		Delegation		***P***value
		**n**	**%**	**n**	**%**	
**Delegation** ***Overall***	**2,063**	**929**	**45.0**	**1,134**	**55.0**	
**Gender**						
Female	926	419	45.2	507	54.8	0.447
Male	1,137	510	44.9	627	55.1	
**Payment status (adults only; n = 1,740)**						
Non-exempt	1,569	793	50.5	776	49.5	
Exempt	171	83	48.5	88	51.5	0.630
**Age groups**						
Under 18 years	282	41	14.5	241	**85.5**	0.001*
Working age (18–64 years)	1,567	789	50.4	778	**49.6**	
Over 64 years	214	99	46.3	207	**53.7**	
**Quintiles of deprivation based on patient population (n = 2,043)**						
Most deprived 1	445	200	44.9	245	55.1	0.988
2	423	192	45.4	231	54.6	
3	483	222	46.0	261	54.0	
4	450	199	44.2	251	55.8	
Least deprived 5	241	108	44.6	134	55.4	
**Smoking cessation signposting (adults only; n = 541)**						
No	196	145	74.0	51	**26.0**	0.001*
Yes	345	191	55.4	154	**44.6**	

*Statistically significant differences in bold*; n = 2,063 unless otherwise stated.

### Patient age and delegation

**Figure 1:**
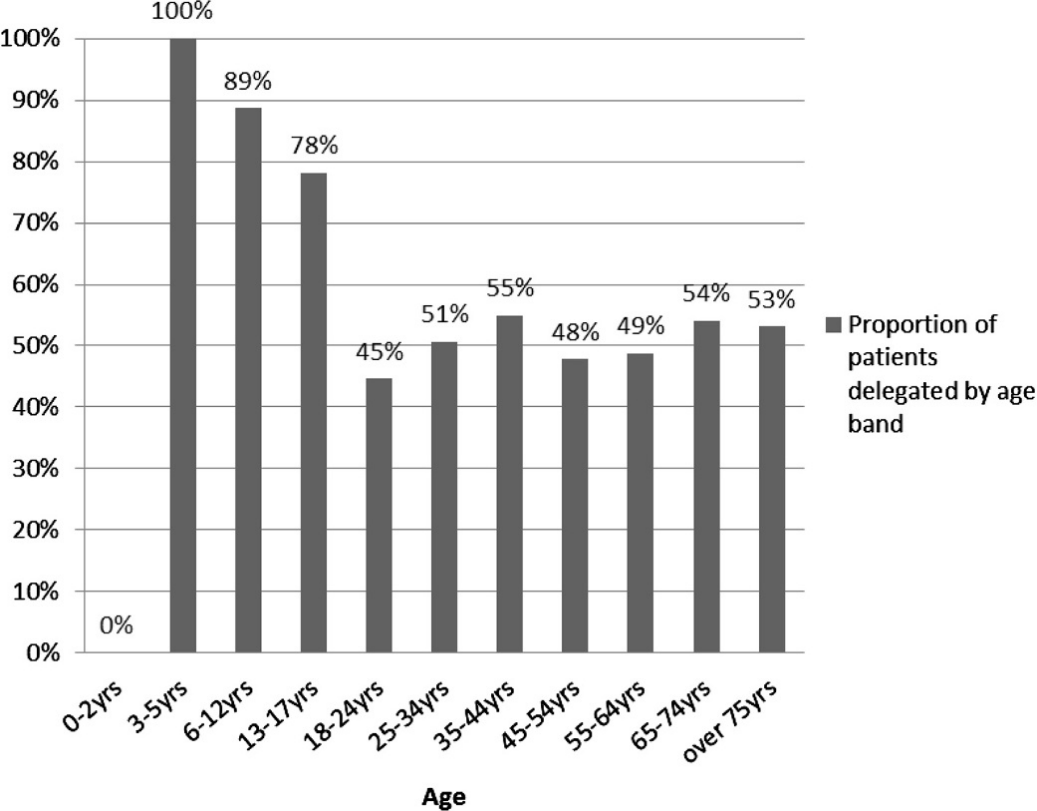
**Proportion of patients delegated by age group.**

The relationship between age and delegation was examined further by 11 age groups (Figure 1), and the findings indicate that a larger proportion of younger patients were delegated compared to older aged patients; this ranged from 100% of 3–5 year olds delegated compared to only 44% of 18–24 year olds. Amongst adult patients, the 35–44 year age-group had the highest level of delegation (55%).

### Treatment type and delegation

**Figure 2:**
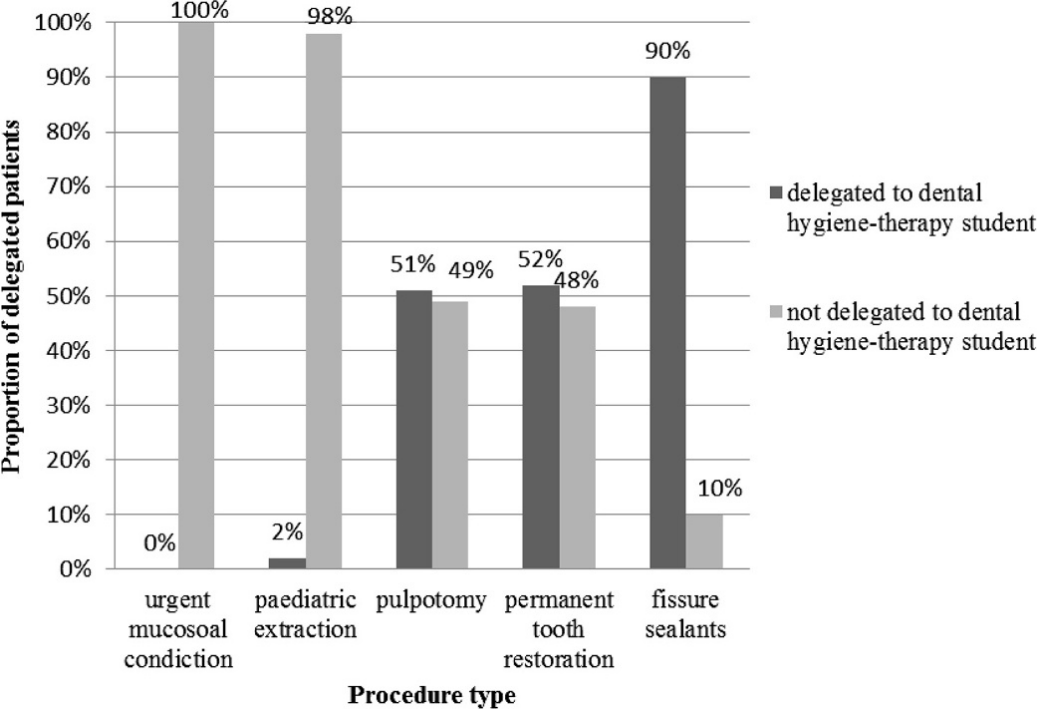
**Rate of treatment delegation from dental students to hygiene-therapy students.**

Overall, 46% of the treatments in the data set analysed were delegated. The procedures most commonly delegated were fissure sealants (90%), restorations (52%), and pulpotomies (endodontic treatment on primary teeth) (51%). The least delegated operations were paediatric tooth extractions (2%) as outlined in Figure 2. Procedures involving management of soft tissue mucosal lacerations or bleeding, classified as urgent, were performed by dental students.

## Discussion

The findings of this study provide insight into the pattern of delegation from dental students to hygiene-therapy students during training, with analysis restricted to the higher level of treatments within the scope of practice of dental hygiene-therapists. The data suggest that, in this educational establishment, 46% of these higher level treatments and 55% of patients receiving this care were delegated by the dental students to dental hygienist-therapy students. Overall, dental hygiene-therapy students delivered a higher proportion of preventative tasks and undertook a significantly higher proportion of care on children and adult smokers. The findings do, however, need to be considered within the context, which is a state-funded primary care educational facility in the South of England where patient charges for adults were not applied. Furthermore, it is important to recognise that the findings are an under-representation of the overall clinical care provided by the dental hygienist-therapists students, as some simple elements of care were not coded by skill mix and attributed to them in the management system.

The high rate of delegation of children (86%) compared with adults (50% of 18–64 years old and 54% of ≥65 years old) could be attributed to a number of factors. First, the more widely accepted and traditional role of the dental hygiene-therapist in children’s care; since the first dental therapists were introduced to work in school dental services in New Zealand [35] the perception that those with therapy training are well suited to caring for children has persisted. Second, given this is an educational establishment, the need for students to gain certain clinical experience undoubtedly plays a role; dental hygiene-therapy students only have the opportunity to treat children at UPDA, whilst dental students do so in other settings. The findings, therefore, demonstrate that hygiene-therapists gain experience in children’s care. This is important as they move forward in their careers as various studies suggest there are gains to be made in patient outcomes and productivity through their utilisation in children’s care [36, 37].

Moving on to the rationale for the delegation rate of adult care, the lower level of delegation amongst adults may be attributed to a number of factors. First, ‘scale and polish’, a common component of adult care, was not coded by provider of care and therefore could not be included in the analysis. This may have reduced the potential for a large number of adult patients who had been delegated for that procedure from being included in the analysis. Second, adult patients may have required more complex overall care, therefore necessitating the additional knowledge and skills of a dentist. Third, dental students at this level need experience of more complex procedures, e.g., endodontic treatment, fixed and removable prosthodontics, and so may be more selective in focusing their clinical time on complex tasks required by patients and delegating routine care.

This patient management system data revealed that preventative care which was well within the scope of a dental hygiene-therapist was delegated at a higher rate than restorative tasks. While still considering prevention, it is noteworthy that the contribution of dental hygiene-therapists to health promotion, including clinical prevention, is considered vital for contemporary evidence-based care [38, 39]; this includes items such as fissure sealants [40]. Furthermore, the experience gained by these students in prevention programmes, therefore, places them in an ideal position to participate in prevention, particularly as policy makers in England are placing greater emphasis on targeted public health programmes [41] and clinical prevention [42]. In countries such as Brazil, oral health technicians, with a similar scope of practice, have found an invaluable place in the provision of public health programmes [14].

These findings suggest that a significantly higher proportion of smokers than non-smokers were delegated to dental hygiene-therapists for clinical treatment. According to the system of practice at UPDA, patients identified as smokers are signposted to smoking cessation services. Dental team members do not provide specific smoking cessation counselling nor are they able to prescribe aids to cessation such as nicotine replacement therapy, etc. The fact that a large number of smokers are treated by dental hygiene-therapy students highlights opportunities for health messages, including more specific smoking cessation support, and thus contributes to the management of common risk factors [43]. Evidence from the US, UK, and Australia suggests that dental hygienists and dental hygiene-therapists can successfully play a role in providing smoking cessation counselling [44–46].

This analysis reveals vital information on the delegation pattern for a range of restorative tasks, which are within dental therapists’ scope of practice, but notably are not as widely performed once qualified [47, 48]. The findings compare broadly with those of Evans et al. [11], who suggest that a significant amount of care (35% of care visits and 43% of clinical time) could be delivered by trained dental hygienists and therapists. However, there is evidence that qualified dental hygiene-therapists in the UK undertake more simple hygiene than therapy work, which is a possible concern as it may lead to de-skilling [48]. It is worth noting that dental hygiene-therapists’ scope of practice supports their working in either role allowing them to deliver both routine restorative work and periodontal care to adults [13], as well as prevention.

This study focused on the recorded clinical experiences gained by both dental students and dental hygiene-therapists when trained together, and thus provides insight into their preparation for future practice. Although the study has limitations due to the inability to analyse total procedures delegated in the 2 year period, it does provide clear quantitative insight to a model of skill mix. Furthermore, this is an educational facility where curricula and learning may play a part in determining who provides what care in the dental team; however, the possibility that these early professional behaviours may influence future professional working patterns should be considered. For the educational facilities, the knowledge of the range and type of patients treated by dental hygiene-therapists, especially risk groups such as smokers, is useful in planning training of the students in health promotion.

Further research is required to understand when and why students in training delegate or refer on particular treatments or patient groups. There is also room to explore how different models of skill mix and delegation rate relate to demand for care.

As mentioned above, one limitation of this study was that not all care was coded by the provider in the patient management system and thus limited the analysis; therefore, it is recommended that all primary care patient management systems should apply codes to indicate which type of operator provided care, so that a greater understanding of skill mix can be gained. This small change would provide additional insight to primary dental care working practices, both current and future.

## Conclusions

The findings suggest that delegation of care to dental hygiene-therapy students in team training with dental students, involved significantly more experience in treating children and adult smokers, and providing preventive rather than invasive care during their clinical training in an integrated educational and primary care service. Educators and planners of dental services seeking to improve and understand the use of dental team skill mix, should consider coding all treatment items in patient management systems, by type of operator, in order to facilitate a wider understanding of the clinical experience and productivity of different members of the dental team in the provision of patient care.

## Abbreviations

DCPs: Dental care professionals
GDC: General Dental Council
NHS: National Health Service
UPDA: University of Portsmouth Dental Academy
DHT: dental hygiene- therapy.
